# Training spatial hearing in unilateral cochlear implant users through reaching to sounds in virtual reality

**DOI:** 10.1007/s00405-023-07886-1

**Published:** 2023-03-11

**Authors:** Chiara Valzolgher, Sabrina Bouzaid, Solene Grenouillet, Julie Gatel, Laura Ratenet, Francesca Murenu, Grégoire Verdelet, Romeo Salemme, Valérie Gaveau, Aurélie Coudert, Ruben Hermann, Eric Truy, Alessandro Farnè, Francesco Pavani

**Affiliations:** 1 Center for Mind/Brain Sciences (CIMeC), University of Trento, Corso Bettini, 31 Rovereto, Trento, Italy; 2 Integrative, Multisensory, Perception, Action and Cognition Team (IMPACT), Lyon Neuroscience Research Center, University of Lyon 1, Lyon, France; 3 Hospices Civils de Lyon, Lyon, France; 4 Neuroimmersion, Lyon, France; 5 Centro Interuniversitario di Ricerca “Cognizione, Linguaggio e Sordità” (CIRCLeS), Trento, Italy

**Keywords:** Spatial hearing, Cochlear implant, VR training, Virtual reality, Reaching, Active listening, Head movements

## Abstract

**Background and Purpose:**

Use of unilateral cochlear implant (UCI) is associated with limited spatial hearing skills. Evidence that training these abilities in UCI user is possible remains limited. In this study, we assessed whether a Spatial training based on hand-reaching to sounds performed in virtual reality improves spatial hearing abilities in UCI users

**Methods:**

Using a crossover randomized clinical trial, we compared the effects of a Spatial training protocol with those of a Non-Spatial control training. We tested 17 UCI users in a head-pointing to sound task and in an audio-visual attention orienting task, before and after each training. <br>Study is recorded in clinicaltrials.gov (NCT04183348).

**Results:**

During the Spatial VR training, sound localization errors in azimuth decreased. Moreover, when comparing head-pointing to sounds before vs. after training, localization errors decreased after the Spatial more than the control training. No training effects emerged in the audio-visual attention orienting task.

**Conclusions:**

Our results showed that sound localization in UCI users improves during a Spatial training, with benefits that extend also to a non-trained sound localization task (generalization). These findings have potentials for novel rehabilitation procedures in clinical contexts.

**Supplementary Information:**

The online version contains supplementary material available at 10.1007/s00405-023-07886-1.

## Introduction

In case of neurosensory deafness, standard interventions often comprise the application of cochlear implants (CI). Although this surgery is indicated for people with bilateral hearing loss, many patients receive only one CI [1, 2]. Using only one CI and listening asymmetrically leads to difficulties in sound localization. Sound localization is poorer with unilateral rather than bilateral CI [3]. Similarly, switching-off one implant in bilateral CI users (BCI) compromises sound localization in the horizontal dimension [4, 5]. Spatial hearing difficulties in unilateral CI users (UCI) have been attributed to the reduced availability of auditory cues. The CI alters auditory cues due to its restricted spectro-temporal processing [6]. In addition, it can modify sound intensity through automatic gain control (AGC) or alter auditory cues through noise reduction strategies or through filters emphasizing higher frequencies [10]. Wearing one CI also minimizes binaural inputs, which are crucial to localize sounds along the horizontal dimension [10–13]. Even when binaural hearing experience is only reduced and not absent, as in the case of bimodal stimulation (e.g., a cochlear implant in one ear and a contralateral hearing aid in the other), sound localization is perturbed [14]. Binaural cues are distorted and compromised by technology difference between device (e.g., the device delay mismatch [15]), while monaural spectral pinna cues are poor or not preserved by hearing devices’ features (i.e., microphone behind the ear) [16].

In this context of impoverished auditory cues, can CI users improve their sound localization skills? In people with normal hearing listening with one ear plugged, sound localization abilities can be trained [17–19]. Pioneering results suggest that this may also be possible in UCI [20] and BCI users [21]. Recently, we showed that a multisensory-motor training can rapidly change sound localization skills in BCI users [22, 23]. Taking advantage of virtual reality (VR) technologies, we promoted active interactions with sound sources through hand-reaching and head movements. We found that such an active exploration of the acoustic environment enhanced sound localization performance in normal hearing adults with one plugged ear [24] as well as BCI users [22]. These findings are in line with recent studies, which showed that CI users and people with hearing deficits can improve their sound localisation ability when head movements are allowed [25, 26]. Most importantly, we reported that training-related benefits can generalize, extending to a sound localization task in which both the response modality and stimulation positions were novel compared to the trained ones [22, 24].

In the present study, we leveraged such VR training protocol based on active interactions with the auditory scene. To test the efficacy of this training in 17 UCI users, we contrasted this Spatial training with a control procedure that did not entail processing of spatial features of the sound (i.e., the Non-Spatial training). Crucially, we compared these two VR trainings in a crossover experimental design, which allow us to test the effect of both training paradigms on each participant. Before and after each training paradigm, we tested participants in a head-pointing to sound localization task, which entails different sound positions and requires localizing sounds using a different effector (head instead of hand). In addition, to probe for training benefits when implicit sound localization is required, we tested participants in an audio-visual attention orienting task, in which they were asked to judge the elevation of a visual stimulus while listening a sudden sound.

## Methods

### Participants

Twenty UCI participants were recruited to participate in the study. Sample size was based on two previous experiments addressing a similar research question with an identical experimental design, but with different populations (normal hearing: [24]; bilateral CI users: [22]). Three participants were excluded from the analyses (one did not complete the second visit, one abandoned after wearing the Head Mounted Display (HMD), one did not fully match the inclusion criteria; mean age for the included participants was 45.8 years, SD = 16.4; 8 males, 13 right-handed). Three participants asked to interrupt the Spatial VR training because of fatigue (participants 5, 15 and 20 performed 104, 104 and 131 trials out of 156, respectively).

All participants were recruited at the ORL department of the civil hospital Edouard Herriot (HEH) in Lyon (France), and tested in a dedicated room inside the HEH premises. All had normal or corrected-to-normal vision and reported no movement or vestibular deficit, nor neurological or psychiatric history. Anamnestic and clinical data for individual UCI participants are provided in Table 1. During the experiment, participants used their daily sound processor settings (see Table 2 for details about CI model, processor strategy and microphone settings) and 10 of them wore hearing aid on the non-implanted ear. We let each participant perform the task with or without their hearing device in the ear contralateral to the CI, because we aimed to test their sound localization ability in the context of the acoustic stimulation they usually experience. Accordingly, in Table 2 we reported the pure tone average (PTA) threshold in the ear contralateral to the implant, as measured in the condition in which participants performed the experiment: i.e., with or without hearing aid. We calculated them by computing the average between the thresholds available in clinical record for each subject for 250, 500, 1000, 2000, 4000, 8000 Hz. The study was approved by a national Ethical Committee (Ile de France X, N° ID RCB 2019-A02293-54) and recorded in clinicaltrials.gov (NCT04183348). Before starting the experiment, each participant signed an informed consent.

**Table 1:** Anamnestic and clinical characteristics of CI participants

ID	Gender	Age	Etiology of deafness	Age at deaf diagnosis (y; m)	Age at first hearing aid (y; m)	Age at implantation (right ear) (y; m)	Age at implantation (left ear) (y; m)	Years with one CI
uCI 01	F	35	Unknown	7	9	33	–	2
uCI 02	M	63	Otosclerosis	24	24	60	–	3
uCI 03	F	61	Unknown	32	32	55	–	6
uCI 04	M	48	Radiotherapy/chemotherapy	37	37	–	44	4
uCI 05	M	60	Aminoglycoside treatment	54	55	56		4
uCI 06	F	46	Unknown	5	5	–	31	15
uCI 07	M	64	Otosclerosis	37	43	58	–	6
uCI 08	F	57	Genetic	6	17	50	–	7
uCI 09	F	24	Genetic	1	1	7	–	17
uCI 11	F	24	Unknown	1	1	4	–	20
uCI 12	F	21	Meningitis	0;3	1	–	12	9
uCI 14	F	32	Unknown	16	16	–	30	2
uCI 15	M	24	Unknown	0;4	1	2	–	22
uCI 16	F	60	Unknown	30	41	–	57	3
uCI 17	M	38	Genetic	1	2	33	–	5
uCI 18	M	56	Neonatal resuscitation	0	50	51	–	5
uCI 20	M	66	Meniere’s disease	51	57	58	–	8

**Table 2:** Extended information about participants’ cochlear implants

	**Right**					**Left**				
**ID**	**Brand**	**Processor**	**Strategy**	**Microphone directionality**	**Number of active/total electrodes**	**Brand**	**Processor**	**Strategy**	**Microphone directionality**	**Number of active/total electrodes**
uCI 01	Medel	Rondo	Fs4	NA	9/12	*No hearing aid [Hearing Threshold unaided 120 dB HL]*				
uCI 02	Oticon medical	Neuro 2	Crysalis	Omnidirectional	18/20	*Hearing aid [Hearing Threshold aided 46 dB HL]*				
uCI 03	Medel	Concerto	Fs4P	Omnidirectional	10/12	*Hearing aid [Hearing Threshold aided 39 dB HL]*				
uCI 04	*Hearing aid [Hearing Threshold aided 37 dB HL]*					Cochlear	Cp910	Ace	Scan	20/22
uCI 05	Cochlear	Cp910	Ace	Scan	22/22	*No hearing aid [Hearing Threshold unaided 62 dB HL]*				
uCI 06	*Hearing aid [Hearing Threshold aided 48 dB HL]*					Cochlear	Cp1000	Ace	Omnidirectional	22/22
uCI 07	Cochlear	Cp1000	Speak	Omnidirectional	18/22	*Hearing aid [Hearing Threshold aided 45 dB HL]*				
uCI 08	Medel	Rondo1	Fs4P	Omnidirectional	12/12	*No hearing aid [Hearing Threshold unaided 71 dB HL]*				
uCI 09	Advanced bionics	Naida ci q70	Hires optima.s	Omnidirectional	16/16	*Hearing aid [Hearing Threshold aided 53 dB HL]*				
uCI 11	Cochlear	Co910	Ace	Scan	20/22	*No hearing aid [Hearing Threshold unaided 90 dB HL]*				
uCI 12	*Hearing aid [Hearing Threshold aided 35 dB HL]*					Cochlear	Cp1000	Ace	Omnidirectional	22/22
uCI 14	*Hearing aid [Hearing Threshold aided 40 dB HL]*					Medel	Sonnet	FS4	Natural	12/12
uCI 15	Cochlear	Cp1000	Speak	Omnidirectional	20/22	*No hearing aid [Hearing Threshold unaided 100 dB HL]*				
uCI 16	*Hearing aid [Hearing Threshold aided 69 dB HL]*					Medel	Rondo 2	FS4	Omnidirectional	12/12
uCI 17	Advanced bionics	Naida ci q70	Hires optima.s	Omnidirectional	16/16	*Hearing aid [Hearing Threshold aided 68 dB HL]*				
uCI 18	Cochlear	Cp1000	Ace	Scan	22/22	*No hearing aid [Hearing Threshold unaided 100 dB HL]*				
uCI 20	Cochlear	Cp1000	Ace	Scan	22/22	*No hearing aid [Hearing Threshold unaided 57 dB HL]*				

Note that the thresholds reported refer to PTA in the condition in which participant performed the experiment (with or without hearing aid)

### Study design

The entire experiment was conducted inside VR environment. Participants wore a HMD (resolution: 1080 × 1200 px, Field Of View (FOV): 110°, Refresh rate: 90 Hz) that produced an immersive VR experience: participants always saw a reproduction of the room in which they were located. Importantly, the VR also allowed continuous tracking of their head posture and movements. All sounds were delivered from a real speaker, tracked in 3D space and moved by the experimenter’s hand to pre-determined positions within the VR environment (identical to the methods adopted in [22–24] for use of this VR approach in CI users). Use of an actual sound source in the environment was motivated by the difficulty of creating replicable auditory virtual sounds for people using CIs or hearing aids (although extensive efforts have been made to render virtual acoustic scenes also for these hearing assisted populations [28–30]). The immersive VR gave full control over the multisensory cues of testing environment and sound source position, and permitted to provide audio-visual feedback in case of errors.

**Fig. 1:**
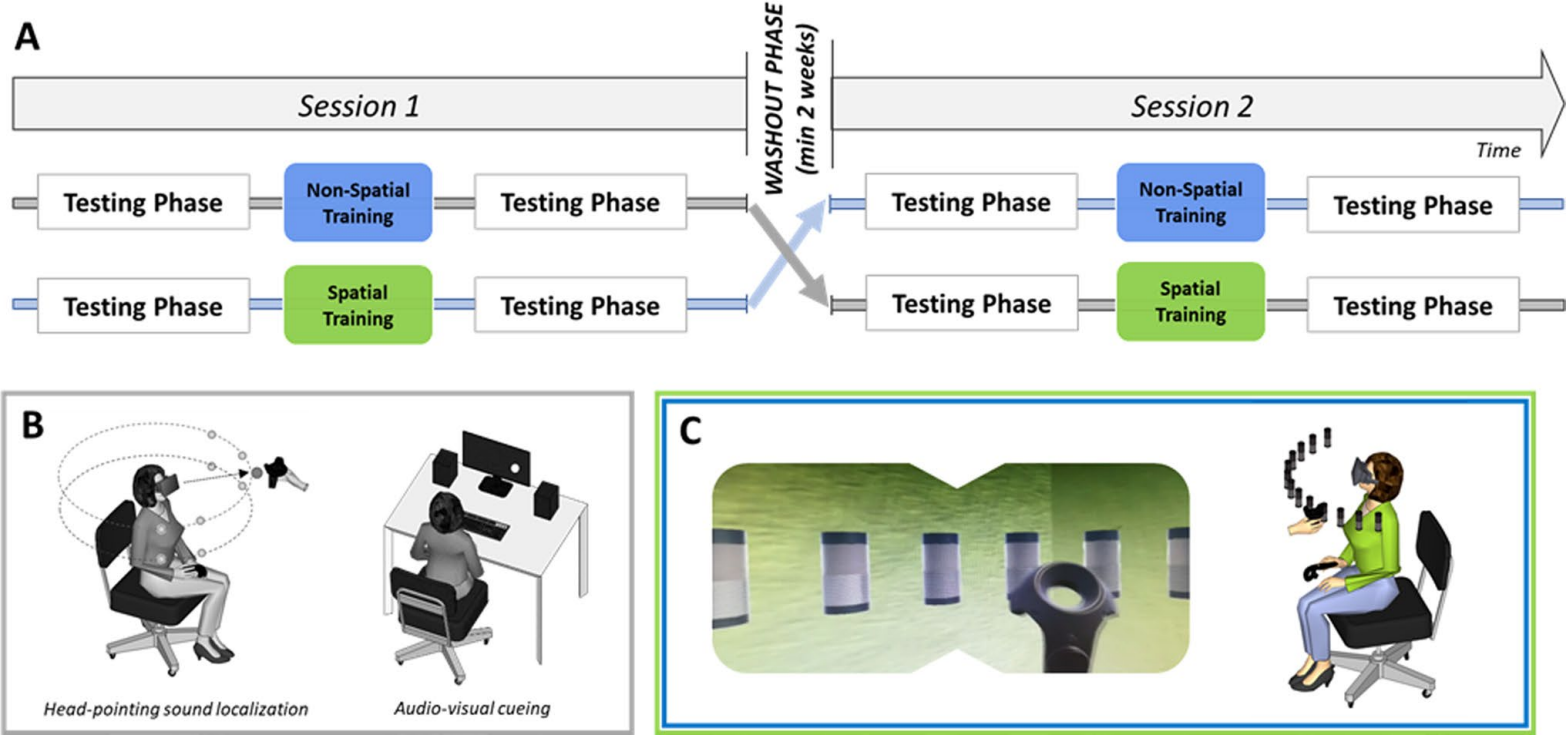
Experimental procedure and setting. **A** Schematic description of the overall crossover design. Each session (Session 1 and Session 2) comprised two testing phases, separated by a training task: Non-Spatial VR in blue and Spatial VR in green. **B** Testing phases. Left: schematic representation of the participant wearing the HMD and holding the VR controller during the head-pointing sound localization task. The grey circles represent the 8 possible positions in which the real loudspeaker could be placed (shown here only for illustration purposes, as no visual cue to sound position was available in the VR environment). They were located 55 cm from the center of the subject’s head, at different azimuth (± 22.5° and ± 67.5° with respect to the midsagittal plane) and vertical positions (5° and − 15° with respect to the plane passing through the ears). Note that the real speaker was never visible in the VR environment. Right: schematic representation of the setting for the audio-visual cueing task (conducted entirely outside VR). **C** Training phase. Left: close-up of the scene as visible inside the HMD from participant’s perspective. The virtual scenario comprised a room, 13 speakers and the VR controller held in participants’ hands. Right: schematic representation of the participant wearing the HMD and holding the VR controller during the training tasks

Participants performed each of the two VR training (Spatial and Non-Spatial) in a within-subject crossover design, in two separate experimental sessions (washout interval was at least 15 days, training order was counterbalanced across participants; see Fig. 1). Before and after each VR training session, participants completed testing phases that comprised two different auditory tasks: a head-pointing localization task and an audio-visual attention orienting task (the only task conducted outside VR).

### Procedures

#### Testing phases

##### Head-pointing sound localization task

 In each trial, a single auditory target (3 s white noise burst) was presented from 8 possible pre-determined positions (5 repetitions each, resulting in 40 trials in each testing phase). The 8 positions were placed at 55 cm from the center of the subject’s head and they varied along the azimuth dimension (± 22.5° and ± 67.5° with respect to the midsagittal plane) and vertical dimension (5° and − 15° with respect to the plane passing through the ears). The variation along the vertical dimension was introduce only to increase variability in the task, and we did not expect training-related changes in this dimension in which sound localization relies on monaural spectra cues. For this reason, we did not analyze performance along the vertical dimension or have hypothesis about participants errors along the vertical plane. While listening the sound, participants were not informed about the pre-determined target positions and were immersed in an empty virtual room (identical size to the real room, i.e., 3.6 m × 3.9 m, height 2.7 m). Participants were instructed to point with their head toward the perceived sound position, as soon as the sound finished, and validate their response using the VR controller they held in their right hand (Fig. 1B). In this specific task, the speaker was always invisible in the VR environment. Notably, while initial posture was identical for all participants and trials, head movements were unrestrained from target onset to the response. The task lasted approximately 15 min.

##### Audio-visual cueing task

 This task aimed to assess to what extent lateralized sound could capture the participant’s audio-visual attention. The task was performed outside VR in the same room of the sound localization task, with participants sat at a desk in front of a computer monitor flanked by speakers. In each trial, a visual disk appeared above or below eye-level (± 1.15°), on the left or right side (128 trials overall, equiprobable across the four possible positions). Participants were instructed to discriminate the vertical position of the disk as fast and accurately as possible, using up/down arrows keys on the keyboard (Fig. 1B). Each disk was paired with a task-irrelevant sound delivered from one of two loudspeakers flanking the screen. The sound was either delivered on the same side as the visual disk (spatially congruent trials) or from the opposite side (spatially incongruent trials), with equal probability. In normal hearing participants, this procedure results in automatic audio-visual orienting of selective attention, i.e., participants are faster and more accurate when responding to visual targets appearing on the same side as the preceding sound [31]. All head movements were restrained by a forehead and chin-rest. The task lasted approximately 10 min.

#### VR training tasks

Participants were immersed in the same virtual room as the head-pointing sound localization task, but saw 13 virtual loudspeakers spanning ± 72° in front space (see Fig. 1C). In each trial, a sound was emitted by a real speaker moved by the experimenter, as in the head-pointing sound localization task (12 repetitions per loudspeakers, total 156 trials). Half of the sounds were amplitude modulated at 2 Hz, the remaining half at 3 Hz. Hence, irrespective of the VR training task (Spatial or Non-Spatial), the stimulation changed unpredictably in location and amplitude modulation on a trial-by-trial basis.

##### Spatial VR training

Participants were instructed to reach the speaker emitting the sound using the VR controller they held in their right hand. The sound lasted until the participant reached and ‘touched’ the correct speaker. If they reached the wrong speaker, the correct loudspeaker started to display concentric red beams that expanded from the correct position to reach, and the sound continued until the correct location was finally reached (a video that illustrates the training tasks is available in 22, http://links.lww.com/EANDH/B44).

##### Non-Spatial VR training

Participants were instructed to identify the amplitude modulation in the target sound, and indicate their discrimination through a reaching movement using VR controller. For fast amplitude-modulated sounds, participants reached in front of them, aiming to touch the invisible virtual button placed above the central speaker. For slow amplitude-modulated sounds, participants reached instead the invisible virtual button placed below the same central speaker. As in the Spatial Training feedback procedure, the sound stopped only when a correct response was provided. If they reached the wrong button, a visual feedback was displayed and the sound continued until the correct button was finally touched. In both trainings, the feeling of touch was induced by making the controller vibrate as soon as it collided with objects (speakers or invisible buttons).

### Statistical analysis

Linear mixed-effect modeling was used for all statistical analyses. Statistical analyses were run using R (version 1.0.143). For the linear mixed-effect (LME) model, we used the R-packages emmean, lme4, lmerTest in R Studio [32, 33]. The R-package car was used to obtain deviance tables from the LME models. For further details about “Materials and method”, see [22] and Supplementary Results for details on each of the analyses described below.

To study head movements, we extracted three dependent variables: number of head rotations, head-rotation extent and head-rotation bias [18]. All the variables concern head rotations around the vertical axis. To calculate the number of head movements, we counted all the detected peaks of velocity in the head trace expect for movements smaller than 2°, which were removed to exclude movements which are not indicators of spontaneous head intentional movements and not related to the task (i.e., micro-postural movements). To calculate head-rotation extent, we sum the absolute value of the rightward and leftward head rotation around the vertical axis extremity, while to calculated head-rotation bias we computed the signed sum of these two values.

## Results

### VR training

#### Performance

**Fig. 2:**
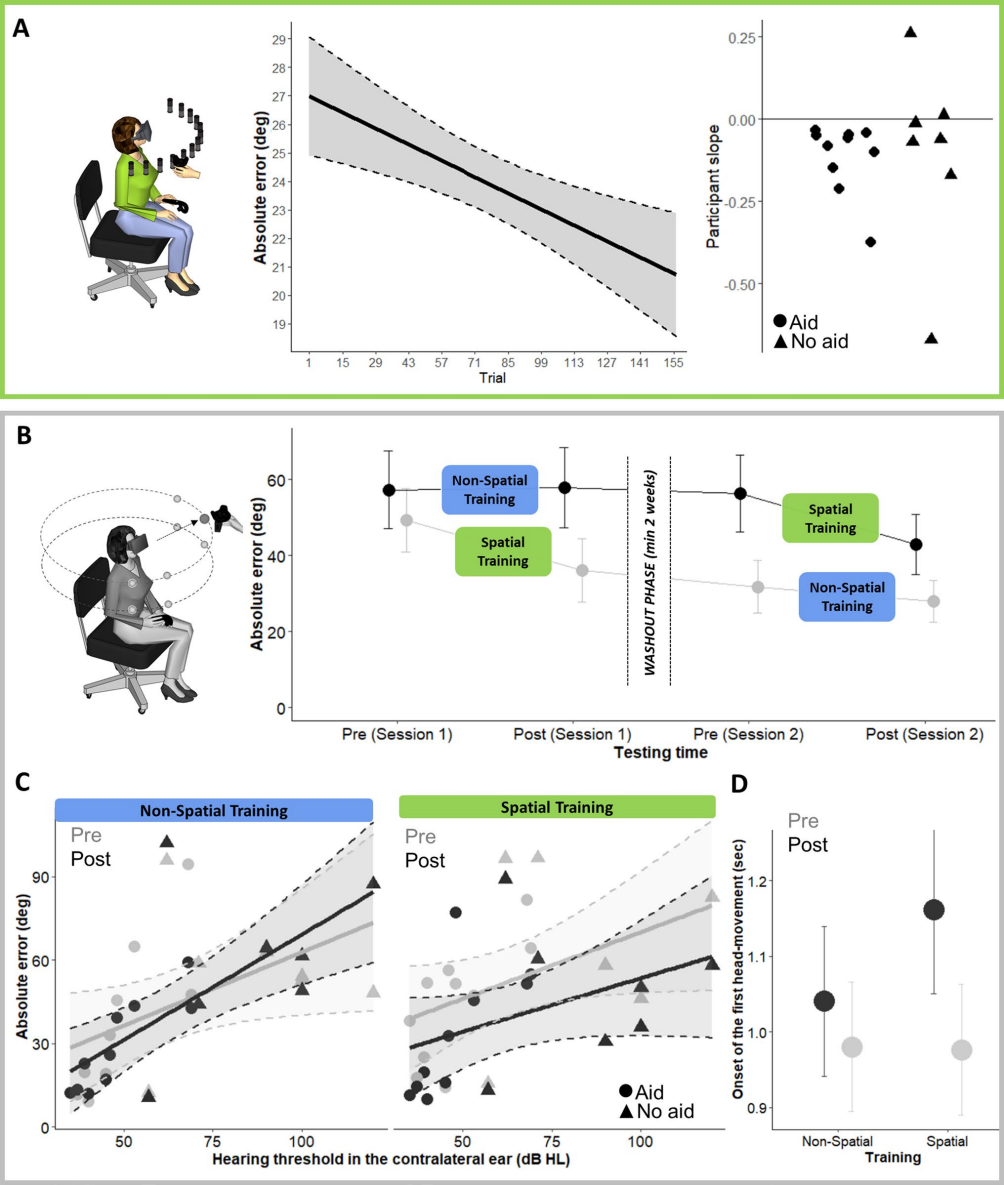
Sound localization performance. **A** Absolute error along azimuth, as a function of trial in the Spatial training. Linear regression (solid line) with 95% confidence intervals (dashed lines). To the left, slope for each participant extracted from the LME model used in the analysis. **B** Absolute localization across the four testing sessions of the experimental design, separately for participants who completed the Spatial training on session 1 (grey) or session 2 (black line). Error bars represent standard errors. **C** Absolute localization error along azimuth dimension as a function of training (Spatial: right and Non-Spatial: left), phase (Pre: grey and Post: black) and hearing threshold in the contralateral ear (x axis). **D** Onset of the first head movements in seconds as a function of phase (pre-training in black and post-training in grey) and trainings. Error bars represent standard errors. In **A** and **C**, circles represent participants who wore hearing aid in the contralateral ear (*N* = 10) and triangles who did not have hearing aid (*N* = 7)

The spatial discrepancy between the stimulated and the reached speaker (i.e., absolute localization error in azimuth, calculated as difference in absolute value between speaker and response position in azimuth in degrees) was on average 24.0 degrees (SD = 14.0), with a numerical (but not-significant) bias toward the side contralateral to the CI (− 2.6°, SD = 7.6; *t*-test against zero: *t*(16) = − 1.40, *p* = 0.19). Importantly, absolute localization errors decreased across trials (*X*^2^ (1) = 4.37, *p* = 0.04), proving that participants improved localization abilities during the Spatial training (Table S1). Figure 2A shows changes in sound localization performance during the Spatial VR training across successive trials. The left panel depicts the overall change across participants; the right panel shows the value of the slope of the regression line for each individual participant, grouped as a function of whether they used a hearing aid in the ear contralateral to the CI or not. For all participants except two the slope was negative, i.e., their performance improved across trials.

We analyzed the influence of hearing asymmetries in hearing thresholds (PTA) between the implanted and non-implanted ear on errors during the Spatial training. We found that the larger the asymmetry (i.e., the higher hearing thresholds in the contralateral ear), the larger the localization error (*X*^2^ (1) = 75.55, *p* < 0.001). Moreover, the bias toward the contralateral ear emerged as a function of hearing asymmetry (*X*^2^ (1) = 4.26, *p* = 0.04). Yet, individual asymmetries in hearing thresholds did not modulate the amount of improvement across time during the Spatial training (all ps > 0.11; see Table S2).

Performance in the Non-Spatial training was near ceiling for all participants (mean number of errors = 1.5%). During the Non-Spatial training, participants were also faster in completing the trial compared to the Spatial training (Non-Spatial: mean ± SD = 2.5 ± 2.1 s; Spatial training: mean ± SD = 16.1 ± 10.2 s; *t* (16) = 11.41, *p* < 0.001 on paired *t*-test).

#### Head movements

Head rotations were overall more frequent in the Spatial (6.51 ± 3.15) compared to the Non-Spatial VR training (1.5 ± 1.0, *W* = 152, *p* < 0.001 on Wilcoxon signed rank test). Likewise, the extent of head rotation movements was larger during the Spatial (123.9° ± 64.0°) compared to the Non-Spatial VR training (15.2° ± 45.2°, *W* = 153, *p* < 0.001 on Wilcoxon signed rank test). Finally, head rotations were more biased toward the side contralateral to the CI during the Spatial (− 27.5° ± 35.8°) compared to the Non-Spatial training (− 0.8° ± 7.8°, *W* = 17, *p* = 0.003 on Wilcoxon signed rank test).

**Fig. 3:**
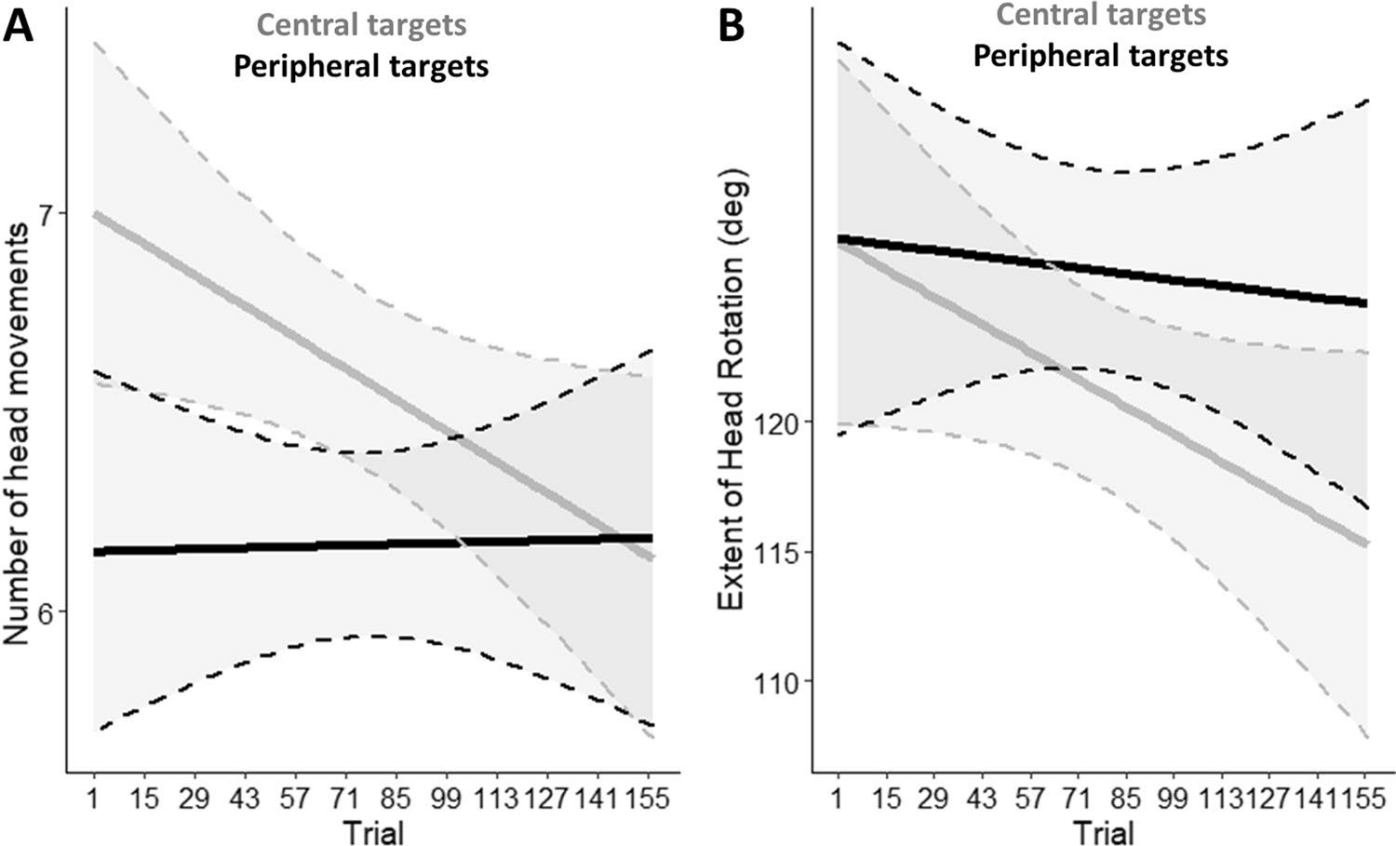
Head rotation during the Spatial training. **A** Number of head movements across trial repetition as a function of sound eccentricity (central positions in grey and peripherical positions in black). **B** Extent of head rotation across trial repetition as a function of sound eccentricity (central positions in grey and peripherical positions in black)

During the Spatial VR training, we observed that UCI users adapted their spontaneous head movements as a function of sound eccentricity as training trials progressed. Specifically, number of head rotations (*X*^2^ (1) = 6.22, *p* = 0.01) and head rotation extent (*X*^2^ (1) = 10.06,* p* = 0.002) diminished as a function of trial repetition, specifically when sounds were emitted by central sources (shown in grey in Fig. 3). This is compatible with participants requiring progressively fewer head movements and smaller extent of head rotation to identify central targets over time. We also observed that participants head-rotation bias changed as a function of sound side (ipsilateral vs. contralateral with respect to the CI, *X*^*2*^ (2) = 1125.33, *p* < 0.001). When the sound was delivered on the same side as the CI, head rotations were markedly biased toward the non-implanted side (− 46.7° ± 32.6°), as if participants aimed to exposed their CI to the sound energy. By contrast, this biased exploration was not evident for sounds delivered on the side opposite to the CI (− 4.2° ± 38.2°) (see Tables S3 and S4).

### Effects beyond the trained task

#### Head-pointing to sounds

##### Performance

The Spatial VR training improved performance (i.e., decreased absolute error in azimuth) more than the Non-Spatial training, irrespective of the session in which it was completed (Spatial—pre: 52.6° ± 26.2°; post: 39.3° ± 23.5°; Non-Spatial training—pre: 43.8° ± 27.4°; post: 42.0° ± 27.6°, *X*^2^ (1) = 23.36, *p* < 0.001; see Fig. 2B). Interestingly, participants who performed the Spatial VR training in the first session (*N* = 9) maintained the improvement after the 2-week washout: errors in the pre-training phase were smaller on session 2 compared to session 1 (*X*^2^ (1) = 94.86, *p* < 0.001).

As documented above, individual asymmetries in hearing thresholds between the implanted and non-implanted ear influenced performance: the higher the threshold in the contralateral ear, the higher the error (*X*^2^ (1) = 462.29, *p* < 0.001). The effect of Spatial training was higher for participants who had higher threshold, as shown in Fig. 2C (*X*^2^ (1) = 59.30, *p* < 0.001) (see Table S5). Participants who wore a hearing aid at ear contralateral to the CI are indicated by circles in Fig. 2.

Trainings also influenced the response bias in sound localization (i.e., the signed error). In the pre-training session, no overall bias toward the side contralateral to the CI was measured (− 1.1°, SD = 39.2; *t*-test against zero: *t*(16) = 0.12, *p* = 0.91). Yet, when the response bias was studied as a function of hearing asymmetry we found that, after each of the training, the participants’ responses changed. The Non-Spatial training increased the bias toward the side of the CI, especially for participants with higher hearing asymmetry (*X*^2^ (1) = 4.18, *p* = 0.04). Conversely, the Spatial training decreased the bias for all participants, especially for participants with higher level of hearing asymmetry (*X*^2^ (1) = 24.97, *p* < 0.001, see Table S6 for further details about the analysis).

To further examine the effect of training on head-pointing to sounds, we analyzed the direction of the first head-rotation in each trial. This measure captures the immediate orienting response toward the sound. We found that participants discriminated the side of sounds source (ipsilateral: 24.09 ± 39.42; contralateral; − 17.18 ± 47.19, *X*^2^ (1) = 58.84, *p* < 0.001). Their accuracy in discriminating sources’ side increased selectively after the Spatial training as compared to the Non-Spatial one (*X*^2^ (1) = 3.09, *p* = 0.05). Another head-movement variable worth considering is the onset of the first head movement of correct responses, which has been used as indicator of the ability to discriminate the side of the sound direction [22, 34]. Irrespective of VR training type, onset of the first head movement decreased after training (*X*^2^ (1) = 7.00, *p* = 0.008). Yet, this reduction was more pronounced after the Spatial (before: 1.16 ± 0.46; after: 0.98 ± 0.35; *t* = 7.36, *p* < 0.001), as compared to the Non-Spatial training (before: 1.04 ± 0.41; after: 0.98 ± 0.35; *t* = 2.65, *p* = 0.008; *X*^2^ (1) = 11.92, *p* < 0.001). This reduction was more pronounced for participants who have higher asymmetry (*X*^2^ (1) = 22.08, *p* < 0.001) (Fig. 2D, see Table S7).

##### Head movements

In order to describe changes in head movement after training, we measured number of head rotations, head-rotation extent, head-rotation bias and direction of the first head movements during the sound (see Analysis for a description of these variables). We report here the main findings, but see Supplementary Materials for further details (Table S8).

**Fig. 4:**
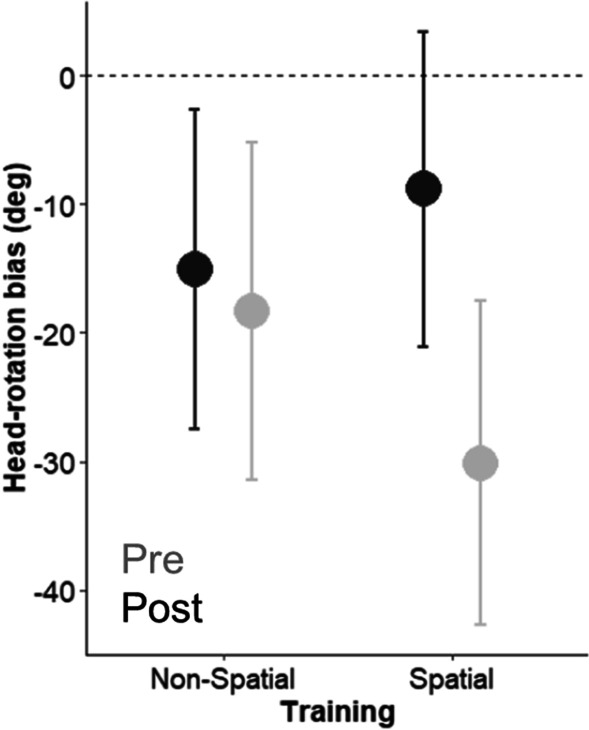
Head-rotation bias during the Head-pointing to sounds task, as a function of training (Spatial Training and Non-Spatial training) and Phase (Before training in black and post-training in grey). Error bars represent standard errors

Participants changed their head-related behavior after each of the training. In the post-training session, they increased the number of movements (Pre: 1.90 ± 0.65; Post: 2.06 ± 0.52, *X*^2^ (1) = 5.16, *p* = 0.02) and increased head-rotation extent (Pre: 99.21 ± 48.77; Post: 101.35 ± 48.39, *X*^2^ (1) = 3.78, *p* = 0.05). Importantly, only after the VR Spatial training they turned their heads toward the contralateral side of the implant to bring the implanted ear toward the sounds: head-bias toward the contralateral space increased more after the Spatial (Pre: − 8.84 ± 50.54; Post: − 30.11 ± 51.05, *t* = 5.42, *p* < 0.001) compared to the Non-Spatial training (Pre: − 15.07 ± 51.05; Post: − 18.35 ± 54.20, *t* = 0.84, *p* = 0.40,* X*^2^ (1) = 10.48, *p* = 0.001) (Fig. 4).

Head movements also changed as a function of hearing asymmetry. Participants with greater hearing asymmetry increased their head-rotation extent after the Spatial compared to Non-Spatial VR training more than participants with less hearing asymmetry (*X*^2^ (1) = 18.03, *p* < 0.001). Likewise, they directed their first head movement toward the contralateral space after the Spatial, but not after the Non-Spatial VR training (*X*^2^ (1) = 15.35, *p* < 0.001).

##### Audio-visual attention orienting task

When people with normal hearing are asked to make a visual discrimination (here on the elevation of the visual target, up vs. down), they are faster when the visual target is preceded by a sound located in the same (congruent) vs. opposite side of the space (incongruent) [35, 36]. This well-documented behavioral effect reveals to what extent a sound can capture auditory and visual attention to its location in space, resulting in perceptual processing benefits across sensory modalities. Notably, UCI participants tested in the present study did not benefit of the congruency between the sound and the visual target, as revealed by the absence of a congruency effect (*X*^2^ (1) = 1.03, *p* = 0.31), nor this effect emerged after training (*X*^2^ (1) = 0.41, *p* = 0.52). The only change between pre- and post-training session was in the overall speed of the response, irrespective of the congruency between the sound and the visual target (*X*^2^ (1) = 9.73, *p* = 0.002), which is compatible with a practice effect. Intriguingly, the difference in reaction time between congruent and incongruent conditions (often termed audio-visual cueing, [35, 36]) changed as a function of hearing asymmetry: the lower the asymmetry, the higher is the cueing effect. After both training paradigms, audio-visual cueing increased specifically for people who had higher asymmetry (*X*^*2*^ (1) = 4.49, *p* = 0.03, see Table S9).

## Discussion

We observed that UCI users can improve their sound localization abilities, despite the substantial impoverishment of the available auditory cues. Thus, acoustic space perception improvement is possible also for people using a single CI, at least in the experimental context we have examined. Specifically, we showed that sound localization of UCI users can improve across trials while engaged in a Spatial VR training and that error reduction extended beyond the trained task. Localization errors decreased after training, as compared to before training, and this decrement (about 13°) was greater after the Spatial compared to the Non-Spatial VR training. Further analyses revealed that hearing asymmetry (as described by PTA at the non-implanted ear) modulated training benefit. Generalization effect of Spatial training was more pronounced for participants with higher hearing asymmetry (i.e., higher hearing threshold at the contralateral ear). Finally, the Spatial VR training had no impact on the audio-visual orienting task, which involves the ability to localize sounds sources indirectly. A possible explanation for the lack of this effect is that active listening was prevented during this task as, for experimental reasons, it was performed using a chin-rest. Although this test represents a firm attempt to test the generalization effect, further studies are needed to investigate the transferability of training effects to tasks in which the ability to localize sounds is implicitly involved.

A previous study by Luntz and colleagues already suggested that training UCI spatial hearing skill is possible, but this early report suffered from methodological limitations. They tested only few participants (*N* = 9) and the spatial positions of targets did not vary between the test and training situations [20]. The present study represents a step forward compared to previous literature because a larger number of UCI users were involved and, most importantly, we tested the effects of training also in a different sound localization task to assess generalization. Furthermore, in the present study we adopted a within-subject experimental design, which gave us the opportunity to directly compare the effects of the experimental training with that of the control training in the exact same participants. We also demonstrated the possibility of improvement using a training of only 156 trials, with is considerably shorter compared to previous studies (four to eight training sessions over a period of 6.5 weeks in [20]; or 8 sessions spanning over 4 weeks as in [23]).

Interestingly, our short training produced effects also in a sound localization task entailing different sound positions, a different response method (i.e., use of the head as pointer instead of reaching sound sources using the hand), and less visual cues available (i.e., potential sounds sources invisible during the test). The difference between the trained task and the test task (i.e., head-pointing to sound) is clearly evident also in the different performance achieved by the participants in the two sound localization procedures. While in the trained task, the absolute error for participants in the Spatial training group was 24° on average, in the test task, they started from an average absolute error of 52.6° to achieve a performance of 39.3° at the end of training. This difference is likely the consequence of the different priors about sound position in the two procedures: during training, all possible sound positions were visually identified, whereas during test, no visual cue helps participants to locate the sound sources.

This result highlights the importance of assessing generalization effect when testing the efficacy of training protocols (see also [37]). In addition, it encourages to pursue testing objectives which deviate even more from the training task, in order to fully examine the potentials of the generalization processes (e.g., using words or syllables as stimuli, see [23]). Notably, our crossover experimental design provides initial evidence of relatively long-lasting effects. Participants who performed the Spatial VR training in the first session maintained the improvement after the 2-week washout. This finding corroborate recent evidence in BCI users that also documented a persistent effect after 2 weeks [22], and prompts the implement longitudinal experimental designs to test this aspect thoroughly in future studies. Despite these encouraging results, it is important to note that the uCI users we tested still showed a limited sound localization ability (their errors are still around 40° after the training). Hence, it should be considered as a further opportunity for improvement, but not an alternative to other solutions that could improve spatial hearing (e.g., bilateral implantation).

Given the large interindividual variability in terms of hearing experience, we investigated if hearing asymmetry influenced sound localization performance and training effects. We observed an increase in the effectiveness of the Spatial training for UCI users with higher levels of hearing asymmetry. This finding supports the idea that it is possible to improve localization of sounds even when auditory cues available are primarily monaural intensity cues, and opens the possibility to offer a similar training to people with unilateral hearing loss. Since the larger training effectiveness was documented in individuals who primarily listen monaurally using their CI, rather than individuals with bimodal experience providing binaural cues, this finding suggest that our training primarily changed the way in which participants exploited the available intensity monaural cues (but see [22] for evidence showing the efficacy of the Spatial training BCI users). In the present study, however, the analyses that examined the role of hearing asymmetry were exploratory. The recruitment of participants was not conducted taking this aspect into account, as it was not included in our original research questions, hence we could not fully control this dependent variable (i.e., hearing asymmetry level). For this reason, we investigated asymmetry along a continuum, considering for each participant the hearing threshold at the ear contralateral to the CI. Future studies should address this aspect more systematically, for instance by manipulating the degree of asymmetry of the participants’ hearing or by considering separately patients with effective bimodal experience (i.e., CI plus an effective hearing aid) and patients with a clear unilateral hearing loss using a CI. Furthermore, even if it was beyond the scope of the present work, a further element which may play a role when training acoustic space perception is deafness onset. Investigating this aspect remains a key question for future research.

A further contribution of the present work concerns the study of head movements’ behavior. First, during training, participants requiring progressively fewer head movements to perform the task and reduced the extent of their head rotation when responding to central targets. This corroborates the observation of a trial-by-trial improvement, that we described above in terms of progressive reduction of performance errors. Second, after training, head movements changed between the pre- and post-training measurements. When we focused on the first head-movement onset during the head-pointing to sounds test, we observed that the correct direction of the sound was identified faster after the Spatial compared to the Non-Spatial VR training. Third, participants also started to spontaneously implement novel head-movement behaviors after the training. Specifically, they increased the number of movements and explored a larger portion of space with the head. This was particularly evident after the Spatial VR training, hinting at the possibility that they moved the head strategically to bring their CI toward the sounds. This strategy might have favored the extraction and use of monaural intensity variation at the CI—pointing again to an advantage of the Spatial training mostly related to the use of monaural cues available at the unilateral CI. This strategy has been already documented in previous studies testing people with normal hearing in monaural listening conditions [18, 24]. These findings highlight the importance of moving the head and engaging in active and exploratory listening behavior when aiming to improve sound localization abilities [25, 26, 38, 39] and to foster relearning processes [24]. They also point to the importance of measuring head movements when assessing sound localization skills, and the notion that promoting head-orienting strategies may play a key role in protocols aimed at training sound localization.

## Conclusion

Using a novel VR training based on reaching to sounds, audio-visual feedback and free head movements during listening, we documented that training sound localization ability in UCI users is possible. While these observations emerged in laboratory setting, they have direct translational implications for the clinical context because the observed improvements did not result from changes in hearing settings and hearing thresholds of the participants. Instead, they were likely the result of recalibration processes and self-regulatory behavior, triggered by a combination of multisensory feedback and actions directed to sounds (with the hand and the head). In turn, these allowed participants to better exploit the residual auditory cues when processing auditory space.

## Supplementary Information

Below is the link to the electronic supplementary material.

Supplementary file1 (DOCX 54 KB)

## Data Availability

Data can be retrieved osf.io/mbshq.
