# Supplementary material for: Training spatial hearing in unilateral cochlear implant users through reaching to sounds in virtual reality
Source: Eur Arch Otorhinolaryngol. 2023 Mar 11;280(8):3661–72. doi: 10.1007/s00405-023-07886-1 (PMC10313844; doi:10.1007/s00405-023-07886-1)
Supplement: Supplementary file 1 — Supplementary file1 (DOCX 54 KB) [file 405_2023_7886_MOESM1_ESM.docx]

**Training spatial hearing in unilateral cochlear implant users through reaching to sounds in virtual reality**

**Chiara Valzolgher PhD ^1,2^, Sabrina Bouzaid ^1^, Solene Grenouillet ^1^, Julie Gatel ^4^, Laura Ratenet ^4^, Francesca Murenu ^1^, Gregoire Verdelet ^1,5^, Romeo Salemme ^1,5^, Valerie Gaveau PhD ^1^, Aurelie Coudert MD PhD ^4^, Ruben Hermann MD PhD ^4^, Eric Truy MD PhD ^4^, Alessandro Farnè PhD ^1,2,5^, Francesco Pavani PhD ^2,1^**

1. Integrative, Multisensory, Perception, Action and Cognition Team (IMPACT), Lyon Neuroscience Research Center, University of Lyon 1, France

2. Center for Mind/Brain Sciences (CIMeC), University of Trento, Italy

3. Department of Psychology and Cognitive Sciences (DiPSCo), University of Trento, Italy

4. Hospices Civils de Lyon, Lyon, France

5. Neuroimmersion, Lyon, France

*Correspondence:

Chiara Valzolgher

Corso Bettini, 31 Rovereto, Trento, Italy

chiara.valzolgher@unitn.it

**Supplementary Results**

**VR Training**

*Performance*

The analyses that examined changes in performance during the Spatial VR training included 2523 observations (recall that three participants asked to interrupt the Spatial VR training because of tiredness, hence they completed 104, 104 and 131 trials out of 156, respectively).

Since participants responded by reaching to discreet positions (i.e., visible loudspeakers), we analysed changes in performance by considering the discrepancy between the correct and the reached loudspeaker. Absolute errors in terms of speaker position across successive trials were analysed using a GLME model, using the Poisson family for count data. Biases in sound localization were analysed by considering the signed discrepancy (positive values indicate an error toward the CI side, negative values indicate an error toward the non-implanted side) using a LME model with trial number as fixed effect. To account for the variability related to individual participants and session in which the Spatial VR training was completed (first or second), we also included the participant (intercept and slope) and session (intercept) as random effects in the model (Table S1, Analysis I and II).

|  |  | *X^2^* | *df* | *p* |
| --- | --- | --- | --- | --- |
| Absolute error |  |  |  |  |
| Analysis I | trial number | **4.37** | **1** | **0.04** |
| Bias |  |  |  |  |
| Analysis II | trial number | 3.48 | 1 | 0.06 |

**Table S1**

To examine if individual asymmetries in hearing thresholds between the implanted and non-implanted ear influenced performance during the Spatial training effects, we expanded the analyses detailed above to include hearing threshold in the contralateral ear as fixed effect (Table S2, Analysis III and IV).

**Table S2**

|  |  | *X^2^* | *df* | *p* |
| --- | --- | --- | --- | --- |
| Absolute error |  |  |  |  |
| Analysis III | trial number | 3.12 | 1 | 0.08 |
|  | hearing threshold | **75.55** | **1** | **< 0.001** |
|  | trial number*hearing threshold | 0.02 | 1 | 0.90 |
| Bias |  |  |  |  |
| Analysis IV | trial number | 0.59 | 1 | 0.44 |
|  | hearing threshold | **4.26** | **1** | **0.04** |
|  | trial number*hearing threshold | 2.54 | 1 | 0.11 |

*Head movements*

To study head movements, we extracted three dependent variables: number of head-rotations, head-rotation extent and head-rotation bias^[[1]](#footnote-1)^.

We entered number of head-rotations in a GLME model (family = Poisson) with trial number and eccentricity as fixed effect. To account for the variability related to individual participants and session in which the Spatial training was completed (first or second), we also included the participant (intercept and slope) and session (intercept) (Analysis V Table S3).

**Table S3**

|  |  | *X^2^* | *df* | *p* |
| --- | --- | --- | --- | --- |
| Number of head-rotations |  |  |  |  |
| Analysis V | trial number | 2.23 | 1 | 0.14 |
|  | eccentricity | **16.34** | **1** | **< 0.001** |
|  | trial number * eccentricity | 6.22 | 1 | 0.01 |

Furthermore, we run a similar LME model entering head-rotation extent (Analysis VI Table S4). To study head-rotation bias, we run a LME model with trial number and side (ipsilateral or contralateral to the CI) as fixed effect. To account for the variability related to individual participants and session in which the Spatial training was completed (first or second), we also included the participant (intercept and slope) and trial session (intercept) (Analysis VII Table S4).

**Table S4**

|  |  | *X^2^* | *df* | *p* |
| --- | --- | --- | --- | --- |
| Head-rotation Extent |  |  |  |  |
| Analysis VI | trial number | **4.93** | **1** | **0.03** |
|  | eccentricity | **19.41** | **1** | **< 0.001** |
|  | trial number * eccentricity | **10.06** | **1** | **0.002** |
| Head-rotation Bias |  |  |  |  |
|  | trial number | 1.50 | 1 | 0.22 |
| Analysis VII | side | **1125.33** | **2** | **< 0.001** |
|  | trial number*side | 1.49 | 2 | 0.47 |

**Effects beyond the trained task**

*Head-pointing to sounds*

*Performance*

To study the effect of Spatial training on head-pointing sound localization, we entered absolute error (log) in azimuth into a LME analysis with phase (Pre or Post Training), training (Spatial or Non-spatial) and azimuth (recoded as everyone has ci on the right) as fixed effects. We also included participant (intercept and slope) and testing session (intercept) as random effects, to account for the variability across participants and the order of training type. 0.2 % of trial were removed because of lost in data tracking. Additionally, deviant data-points were excluded following quantile-to-quantile plot inspection (4%) (Table S5, Analysis VIII). To further analyse the data, we run also a similar LME analysis considering phase, training and hearing threshold in the contralateral ear as fixed effect. We also included testing session (intercept) as random effects (Table S5, Analysis VIV).

**Table S5**

|  |  | *X^2^* | *df* | *p* |
| --- | --- | --- | --- | --- |
| Absolute error |  |  |  |  |
| Analysis VIII | azimuth | 0.002 | 1 | 0.96 |
|  | training | **34.29** | **1** | **<0.001** |
|  | phase | 0.08 | 1 | 0.77 |
|  | azimuth*training | 0.02 | 1 | 0.90 |
|  | azimuth*phase | 0.59 | 1 | 0.44 |
|  | training*phase | **23.36** | **1** | **< 0.001** |
|  | azimuth*training*phase | 3.64 | 1 | 0.06 |
| Analysis VIV | hearing threshold | **462.29** | **1** | **< 0.001** |
|  | training | 1.66 | 1 | 0.82 |
|  | phase | 0.05 | 1 | 0.22 |
|  | hearing threshold * training | **100.77** | **1** | **< 0.001** |
|  | hearing threshold * phase | **30.50** | **1** | **0.01** |
|  | training * phase | 0.15 | 1 | 0.69 |
|  | hearing threshold * training * phase | **59.30** | **1** | **< 0.001** |

Furthermore, we considered signed error as dependent variable. Positive values indicate errors toward the side of the CI, while negative values indicate errors toward the opposite side of the space. We entered signed error (log) in azimuth into a LME analysis with phase, training and hearing threshold in the contralateral ear as fixed effect. We also included testing session (intercept) as random effects (Table S6, Analysis X).

**Table S6**

|  |  | *X^2^* | *df* | *p* |
| --- | --- | --- | --- | --- |
| Signed error |  |  |  |  |
| Analysis X | hearing threshold | 0.00 | 1 | 0.99 |
|  | training | **14.17** | **1** | **< 0.001** |
|  | phase | 0.77 | 1 | 0.38 |
|  | hearing threshold * training | **14.36** | **1** | **< 0.001** |
|  | hearing threshold * phase | **3.83** | **1** | **0.05** |
|  | training * phase | **16.90** | **1** | **< 0.001** |
|  | hearing threshold * training * phase | **25.36** | **1** | **< 0.001** |

To further document the performance, we studied the direction of the first head rotation as indicator of first orientation toward sounds. We entered the onset of the first head rotation (note that we considered only the rotations toward the correct direction, 1795/2593) in a LME model with phase (Pre or Post Training) and training (Spatial or Non-spatial) as fixed effects. To account for the variability related to individual participants and session in which the Spatial training was completed (first or second), we also included the participant (intercept and slope) and session (intercept) (Analysis XI Table S7). Furthermore, we entered the onset of the first head rotation into similar analyses with phase, training and hearing threshold in the contralateral ear as fixed effect. We also included testing session (intercept) as random effects (Table S7, Analysis XII).

**Table S7**

|  |  | *X^2^* | | *df* | | | *p* |
| --- | --- | --- | --- | --- | --- | --- | --- |
| First Head-rotation |  |  | |  | | |  |
| Analysis XI | training | 0.00 | | 1 | | | 0.96 |
|  | phase | | **7.00** | | **1** | **0.008** | |
|  | phase*training | | **11.92** | | **1** | **< 0.001** | |
| Analysis XII | hearing threshold | | 0.00 | | 1 | 0.95 | |
|  | training | | **28.48** | | **1** | **< 0.001** | |
|  | phase | | 2.26 | | 1 | 0.13 | |
|  | hearing threshold * training | | **32.44** | | **1** | **<0.001** | |
|  | hearing threshold * phase | | 0.28 | | 1 | 0.59 | |
|  | training * phase | | **9.31** | | **1** | **0.002** | |
|  | hearing threshold * training * phase | | **22.08** | | **1** | **<0.001** | |

*Head-movements*

To study training effect on head movements, we analysed number of head-rotations, head-rotation extent, head-rotation bias and the direction of the first head-movement^1^. We entered number of head-rotations in a GLME model (family = Poisson) with phase (Pre or Post Training), training (Spatial or Non-spatial) and azimuth (recoded as everyone has ci on the right) as fixed effects. To account for the variability related to individual participants and session in which the Spatial training was completed (first or second), we also included the participant (intercept and slope) and session (intercept) (Analysis XIII Table S8). Similar LME analysis were run for head-rotation extent, head-rotation bias and the direction of the first head-movement (Table S8, Analyses XV, XVII, XIX). Furthermore, we run also further analysis to investigate the effect of hearing asymmetry on head movement and we considered phase, training and HEARING THRESHOLD in the contralateral ear as fixed effect (Table S8, Analyses XIV, XVI, XVIII, XX).

**Table S8**

|  |  | *X^2^* | *df* | *p* |
| --- | --- | --- | --- | --- |
| Number of head-rotations |  |  |  |  |
| Analysis XIII | azimuth | 1.45 | 1 | 0.23 |
|  | training | 0.02 | 1 | 0.87 |
|  | phase | **5.16** | **1** | **0.02** |
|  | azimuth*training | 1.30 | 1 | 0.25 |
|  | azimuth*phase | 0.05 | 1 | 0.82 |
|  | training*phase | 0.04 | 1 | 0.83 |
|  | azimuth*training*phase | 0.67 | 1 | 0.41 |
| Analysis XIV | hearing threshold | 0.01 | 1 | 0.91 |
|  | training | 0.63 | 1 | 0.43 |
|  | phase | 0.55 | 1 | 0.46 |
|  | hearing threshold * training | 0.81 | 1 | 0.37 |
|  | hearing threshold * phase | 0.006 | 1 | 0.94 |
|  | training * phase | 0.008 | 1 | 0.93 |
|  | hearing threshold * training * phase | 0.03 | 1 | 0.86 |
| Head-rotation Extent |  |  |  |  |
| Analysis XV | azimuth | 0.17 | 1 | 0.68 |
|  | training | 7.24 | 1 | 0.007 |
|  | **phase** | **3.78** | **1** | **0.05** |
|  | azimuth*training | 1.53 | 1 | 0.21 |
|  | azimuth*phase | 0.21 | 1 | 0.65 |
|  | training*phase | 0.15 | 1 | 0.70 |
|  | azimuth*training*phase | 0.007 | 1 | 0.93 |
| Analysis XVI | hearing threshold | 3.09 | 1 | 0.08 |
|  | training | 1.83 | 1 | 0.17 |
|  | phase | 0.00 | 1 | 0.95 |
|  | hearing threshold * training | 0.18 | 1 | 0.68 |
|  | hearing threshold * phase | 0.47 | 1 | 0.50 |
|  | training * phase | **17.31** | **1** | **<0.001** |
|  | hearing threshold * training * phase | **18.03** | **1** | **<0.001** |
| Head-rotation Bias |  |  |  |  |
| Analysis XVII | azimuth | **17.07** | **1** | **<0.001** |
|  | training | **10.48** | **1** | **0.002** |
|  | phase | 0.71 | 1 | 0.40 |
|  | azimuth*training | 0.04 | 1 | 0.84 |
|  | azimuth*phase | 0.37 | 1 | 0.54 |
|  | training*phase | **10.48** | **1** | **0.001** |
|  | azimuth*training*phase | 0.04 | 1 | 0.84 |
| Analysis XVIII | hearing threshold | 1.64 | 1 | 0.20 |
|  | training | 1.09 | 1 | 0.30 |
|  | phase | 1.19 | 1 | 0.28 |
|  | hearing threshold * training | 3.71 | 1 | 0.05 |
|  | hearing threshold * phase | 0.76 | 1 | 0.38 |
|  | training * phase | 0.04 | 1 | 0.84 |
|  | hearing threshold * training * phase | 0.84 | 1 | 0.36 |
| First head- rotation |  |  |  |  |
| Analysis XIX | azimuth | **13.08** | **1** | **<0.001** |
|  | training | **28.74** | **1** | **<0.001** |
|  | phase | 0.09 | 1 | 0.77 |
|  | azimuth*training | 0.18 | 1 | 0.67 |
|  | azimuth*phase | 0.33 | 1 | 0.56 |
|  | training*phase | 0.14 | 1 | 0.71 |
|  | azimuth*training*phase | 0.70 | 1 | 0.40 |
| Analysis XX | hearing threshold | **4.89** | **1** | **0.03** |
|  | training | 0.00 | 1 | 0.98 |
|  | phase | **10.66** | **1** | **0.001** |
|  | hearing threshold * training | 3.22 | 1 | 0.07 |
|  | hearing threshold * phase | **12.68** | **1** | **0.003** |
|  | training * phase | **14.35** | **1** | **<0.001** |
|  | hearing threshold * training * phase | **15.35** | **1** | **<0.001** |

*Audio-visual attention orienting task*

To examine the effects of our training protocols on the audio-visual attention orienting task, we entered the response time (milliseconds) into a LME analysis with phase, training and congruency between sound and visual target position (congruent or incongruent). Note that we consider only the correct responses (98%). Again, participant and session (intercepts) were treated as random effects (Table S9, Analysis XXI). To further analyse the data, we run a similar LME analysis considering audio-visual advantage as dependant variable (i.e., the difference in response time between incongruent and congruent conditions) and phase, training and hearing threshold in the contralateral as fixed effect (Table S9, Analysis XXII).

**Table S9**

|  |  | *X^2^* | *df* | *p* |
| --- | --- | --- | --- | --- |
| Reaction time |  |  |  |  |
| Analysis XXI | congruency | 1.03 | 1 | 0.31 |
|  | training | 0.37 | 1 | 0.54 |
|  | phase | **9.73** | **1** | **0.002** |
|  | congruency*training | 0.007 | 1 | 0.93 |
|  | congruency*phase | 0.02 | 1 | 0.88 |
|  | training*phase | 0.42 | 1 | 0.51 |
|  | congruency*training*phase | 0.41 | 1 | 0.52 |
| Analysis XXII | hearing threshold | **4.49** | **1** | **0.03** |
|  | training | 0.06 | 1 | 0.81 |
|  | phase | 2.52 | 1 | 0.11 |
|  | hearing threshold*training | 0.13 | 1 | 0.72 |
|  | hearing threshold*phase | 2.84 | 1 | 0.09 |
|  | training*phase | 0.14 | 1 | 0.70 |
|  | hearing threshold*training*phase | 0.02 | 1 | 0.89 |

1. Valzolgher, C., Verdelet, G., Salemme, R., Lombardi, L., Gaveau, V., Farné, A., & Pavani, F. (2020). Reaching to sounds in virtual reality: A multisensory-motor approach to promote adaptation to altered auditory cues. Neuropsychologia, 149, 107665. doi: 10.1016/j.neuropsychologia.2020.107665. [↑](#footnote-ref-1)
